# Persistence of primitive reflexes associated with asymmetries in fixation and ocular motility values

**DOI:** 10.16910/jemr.17.2.5

**Published:** 2024-08-19

**Authors:** Vicente A. Domingo-Sanz

**Affiliations:** Centro Optométrico Montrull Algemesí, (Valencia), Spain

**Keywords:** eye movement, eye tracking, saccades, vergence, attention, reading, primitive reflexes, Visagraph

## Abstract

This cross-sectional study examined eye movement performance in patients aged 4 to 16 years. Measurements of eye movements were obtained before and after performing therapy for inhibition of four primitive reflexes, asymmetric tonic neck reflex, symmetric tonic neck reflex, labyrinthine tonic reflex and Moro reflex. Subsequently the scores of the four primitive reflexes were compared with the results of five variables: fixation maintenance, % mean saccade size, motility excursions, fixations during excursions and mean duration of fixations. The comparisons showed a significant reduction in evidence of fixation maintenance as well as mean saccade size due to the inhibition of the four primitive reflexes. There was also a significant increase in ocular motility while fixations per saccade and average duration of fixations also decreased significantly. Visual balance between values of both eyes improved in all tests. A device called VisagraphTM III, which measures eye movements, was used for data collection. These results suggest that the oculomotor improvements reflect the involvement of other maturational processes such as the emergence and inhibition of primitive reflexes, the whole reorganization being key to future reading and attentional processes.

## Introduction

There is great interest in the knowledge of eye movements, how they develop and how they are executed. Research usually focuses on physiological origin of the ocular system itself while other characteristics such as functioning of the human body and more specifically, the presence of retained primitive reflexes (PRs) and their influence on oculomotor skills, are not taken into account. Eye movements are a subject of ongoing research as they determine the functioning of possibly the most important sensory system that the human being possesses, which is a microcosm within the brain itself. More is known about oculomotor movements and their cortical control than any other aspect of the motor system (42). PRs are also known as they are observed during the neonatal and infant periods, receeding later as a result of cerebral cortex inhibition and brainstem activity (26). However, it is not known in depth how PRs directly influence two basic visual skills, fixations and saccadic movements as studies relating to both topics are very limited or practically nonexistent. PRs are complex, automated movement patterns mediated by the brainstem which develop during the first weeks of gestation, between 25th and 26th week, are fully present at birth in term newborns and are easily elicited during the first semester of life (8; 56). There are at least 27 different reflex responses that are present at birth or appear in the first weeks and months of life (55). Reflex responses are usually triggered by specific sensory inputs (5). PRs undergo significant evolution during the first year of life and their inhibition appears to be related to an increase in cortical control and the development of normal motor function (7), presumably these innate motor behaviours evolved to facilitate the child's survival (34). Hughlings-Jackson argued the fundamental idea that the higher brain centres elaborated complex processes and evolved from simple sensory and motor elements. He called it “the correspondence of the organism with the environment” and determined the direct relationship between a primitive reflex and its integration into these higher order patterns. The higher centres are adequate for the representation of the body and the conditions to which it responds. Hence, there must be an interaction between body movements, sensory interactions and the world around us (18). Thus, a motor dysfunction can be seen as being caused by the persistence of reflexive and generalized patterns of posture and movement that have not been appropriately inhibited by higher centres of the central nervous system (46). Neurodevelopmental deficits are largely conditioned by disorders of the mechanisms of sensorimotor integration of primary reactions and reflex patterns (47). Thus, PRs, in addition to helping the infant to survive, help them to interact with the environment. With appropriate neonatal development, reflex responses become integrated and allow for the appearance of voluntary motor control through cortically directed action and higher-level cognitive skills (25). The development of motor functions correlates with the integration of PRs (55) and these associated with a higher degree of brain maturation, particularly those associated with frontal brain areas (63), with the level of myelination of the pyramidal tract being the most commonly used criterion to determine the degree of maturation. The process of cerebral myelination begins about three months after fertilization. However, at the time of birth only a few areas of the brain are completely myelinated, such as the brainstem centres that control primitive reflexes, because survival depends on them. Once myelinated, neurons can reach full function and can carry out fast and efficient conduction. (52) If there is any delay in the maturation of the pyramidal pathways, it is reasonable to suppose that there could be a delay in the disappearance of different primitive reflexes. (39. For each primitive reflex, there are typical developmental ages and stages of onset and integration, with most beginning prenatally and integrating within the first year of life. Retention of each PRs beyond the typical stage of integration has been associated with and shown to predict impacts on functional performance and development (25). This suggests the relationship of active PRs with attentional and executive dysfunctions (31). More concretely, persistent reflex abnormalities are very likely to impede optimal acquisition of visual skills (3). Finally, it is worth mentioning that the daily repetition of a series of specific movements allows the inhibition of retained PRs, and it is possible that such inhibition occurs at a much later stage than generally accepted (38).

Neurological examinations that clinicians perform as part of infant monitoring include assessment of the status of PRs to ensure that children reach developmental milestones (16). Their persistence in children and adolescents may indicate diffuse cortical maturational delay and correlate with absent or delayed cognitive and executive development, and more importantly may occur in the absence of any lesion (39). When the process of spontaneous reflex integration is not executed fully, it can disrupt the acquisition of motor skills, related to balance and coordination, and learning (45) and more specifically to the learning of reading and writing (29). Therefore, sequential motor development driven by PR integration will set the right framework for the development of sensory processing, including multisensory processing and sensorimotor integration (62). In conclusion reflex integration is important from the point of view of preparing the child to take on and develop motor functions which support school performance (20).

Many of the characteristics of eye movement behavior during reading appear to be established early in reading development and, for that reason, may be more closely related to early developmental sensorimotor, perceptual, and attentional mechanisms and less to a long-term developmental capacity (58). Children with specific reading difficulties can have problems that extend beyond the range of underlying language-related deficits such as difficulties with balance and motor control, there being a link between reading difficulties and movement control in children. In particular it is highlighted how school performance can be related to the interference of PRs (38). Consequently their uninhibited presence should be a reason for control as it can affect both visual perception and ocular motility (13). Keeping the eyes relatively still, (visual fixation) is not simply a matter of absence of control: it requires as much or more active and precise control than the creation of the movements themselves. In terms of saccade control the movement is so fast and the visual feedback so slow, that we cannot simply stop moving our eyes when we see that the visual target has been reached. The oculomotor system must operate in real time with high control that stops the eyes at the correct time (42). Therefore maintaining fixation is an active task and the inability to hold the eyes in stable fixation could result in increased retinal image motion with blurring and consequent reduced sensitivity to changes in blur or spatial position. Both accommodative and vergence demands and their relationship change during the first few years after birth. As an infant gets older, the demand for accommodation typically decreases while the demand for vergence increases (6). The fovea (parvocellular visual pathway) is important for extracting high-resolution spatial information from letters and words while the parafoveal region (primarily the magnocellular visual pathway) is critical during competent reading to pre-direct subsequent saccade to the next optimal fixation point and enable fluent reading. A dysfunctional magnocellular system induces visual stress conditions that hinder the development of competent, comfortable and sustained reading (64). Saccadic orientation and the use of parafoveal vision to guide saccadic orientation during reading are abilities that are established to a considerable degree in the early stages of reading development (58). In parallel there are significant associations between reading speed, refractive error, and particularly, vergence facility. This suggests, and seems sensible, that students with reading difficulties should initially undergo a full ophthalmological examination in addition to a full binocular vision assessment (49). In summary visual attention can be viewed primarily as a sensorimotor behavior (69), as can stereo acuity, as it is significantly associated with gross and fine motor scores (9). In the case of subjects with attention deficit hyperactivity disorder they usually present abnormal oculomotor behavior, less ability to suppress unwanted saccades and less ability to voluntarily control their fixation behavior (40).

In summary, the presence of certain PRs can affect visual development and academic performance especially in the area of reading (65), leading to abnormal functioning of ocular motility and significantly of saccadic movements (22). In line with the above, its presence can also interfere with visual projection, understanding projection as the pattern of binocular behaviour that determines whether there is an orthophoria or the closest point to it (12).

As far as the body is concerned, it is divided into three axes (Appendix A): X-axis, Z-axis and Y-axis, that help organize eye movements. Meanwhile, three of the four PRs used in this study: tonic labyrinthine, asymmetric tonic neck and symmetric tonic neck reflex, serve to facilitate good postural control. The fourth: Moro reflex, is of key importance in visual organization and stability. It is therefore possible that visual improvement goes hand in hand with motor behaviour and that there is therefore a link between the two.

There is currently no study that cross-checks the data and looks for a possible relationship between PRs and the results obtained from the Visagraph^TM^ III measurement. The aim of this study was to look for the possible correlation between the inhibition of four PRs and the improvement of visual fixation and saccades; to observe whether it additionally increased the balance between the values of both eyes; and finally, to confirm whether the presence of certain PRs could be a reason for alterations or asymmetries in visual skills.

## Methods

### Participants

Our study started by obtaining objective data through the Visagraph^TM^ III, and then stimulated the patients for an average period of 12.2 months in a PR inhibition therapy. Finally, the same visual test was performed again with the same device and under the same conditions to make a comparison and observe if changes in visual skills appeared.

The population initially used to conduct the research was 639 patients. After applying the inclusion and exclusion criteria, retrospective data were collected from a total of 193 patients (30,2%). The study contemplates those patients who started PR integration therapy in our center in the month of November 2015 until those who finished therapy in the month of December 2022. Inclusion criteria were: 1) Patients with binocular dysfunctions; 2) patients with learning difficulties; 3) patients with a combination of the above difficulties. Exclusion criteria were: 1) Patients who had previously performed any type of Vision Therapy in another center; 2) patients who had previously performed any type of PR inhibition treatment in other centers; 3) treatments that did not require a minimum of eight months of motor therapy stimulation; 4) cases in which the percentage of performing the exercises at home was below 80% of days worked; 5) patients over seventeen years of age; 6) patients with ocular pathology; 7) patients under treatment of ADHD with both stimulant (methylphenidate-amphetamine) and non-stimulant (atomoxetine-clonidine-guanfacine) medications; 8) patients with anisometropia; 9) patients with uncorrected refractive errors.

The study was approved by the Medical Ethics Committee of the Hospital Universitario y Politécnico La Fe, Valencia, Spain (Registration number 2023-316-1).

### Materials

The Visagraph^TM^ III is an eye movement monitoring device manufactured by Taylor Associates. It uses infrared emitters and detectors mounted on safety glasses to determine eye position by detecting differential infrared reflections from the cornea, sclera and other anterior ocular surfaces. The goggles transfer the information to a personal computer to which they are connected, processing the information to obtain specific eye movement data (11). Despite its simplicity, the Visagraph^TM^ III provides eye movement reading data comparable to more sophisticated recordings (59). It is a device that compares results with those of a large standardized population (61). The information derived from the visual skills report can serve as a basis for making judgments about subjects' visual/functional competence and, possibly, about the need for visual training to improve their reading efficiency. The visual skills test is often used by vision specialists to gain more information about the patient's control over their visual activity (Visagraph^TM^ III Implementation guide). The visual skills test of the device consists of two parts:

Test 1: In this test the patient had to stare at the X in the centre of the sheet for 15 s without moving their eyes. The patient's ability to do so adequately showed the level of control they had over their visual mechanism. The font used was Times New Roman, bold, size 16.

Test 2: In this test there were two Xs arranged in the centre of the sheet but 123 mm apart. The patient had to look at one of the two Xs and then at the other X and alternate as quickly as possible for 20 seconds. This ocular motility test provided information on the patient's ability to change the fixation position and alternate their eyes in an easy, controlled manner and with reasonable values of saccadic movements. The font used was Times New Roman, bold, size 16. (Visagraph^TM^ III, Test Booklet).

Section "C" of the Diagnostic Assessment of Neurodevelopmental Delay, developed by Sally Goddard Blythe (27), was used to obtain measurements of the four PRs used in this study.

The exercises used for PR Inhibition belonged to two different therapies. For patients up to 7 years of age, the exercises designed by DM Primary Reflexes were used, with a total of 13 potential exercises of which 4 are active, 5 are passive and 4 are with parental help. For children older than 7 years of age, the exercises designed by INPP were used, with a total of 14 potential exercises, of which 12 are active and the remaining 2 are passive. Each therapy is designed for its age group and its use is not recommended for the other population.

### Procedure

In this research 57 patients (29.5%) showed binocular difficulties, 73 patients (37.8%) showed learning difficulties while 63 patients (32.7%) showed a combination of both difficulties. As a first part of the evaluations they had a complete binocular vision evaluation by a specialized Optometrist to determine their visual performance as accurately and objectively as possible, including refraction, as this could be a factor altering the measurements with the reading device (49).

Among the tests performed in the binocular evaluation was the Visagraph^TM^ III, which provides information on fixations and saccadic movements, among others. Once the visual tests were completed, the patient, together with a specialized therapist and over the course of another day, underwent a battery of tests of a strictly motor nature. These provided information on the state of the PRs. As a result of this assessment, the patient received one or more exercises aimed at reorganizing the neuromotor system based on the inhibition of the PRs. Subsequently, bimonthly reviews were performed and, depending on the results, the exercise or exercises were modified, if necessary, to be replicated at home, exercising them daily and uninterruptedly during the therapy period. These exercises were always performed under parental assistance and supervision. The aim of the exercises for patients with retained PRs was to reintegrate these movements as close as possible to the typical sequence of motor development and to organize the motor axes. To achieve adequate reflex inhibition, the movements were to be repeated daily with improvements being understandably slow. The duration of the exercises is variable and varies depending on the age of each patient (10). Once the therapist concluded that the inhibition treatment had come to an end, an Optometrist retested the patient with the Visagraph^TM^ III. These two measurements, the initial one and the final one, were used to compare and obtain objective data and to determine the impact that the PR inhibition therapy had on the visual level. All tests, both visual and motor evaluations, were performed in our centre always under the direction of specialized professionals and under the supervision and presence of the parents.

### Evaluation with the Visagraph^TM^ III

The patients were seated on a fixed chair with their feet always resting on a stool and their backs resting on the back of a chair. The goggles were adjusted for each patient so that they were well fixed without being excessively strained. Next, the interpupillary distance was adjusted monocularly while they fixed their eyes on the Optometrist's nose and covered the opposite eye, then the same operation was repeated with the other eye. They were then instructed not to touch the glasses until the test was completed. The two sheets used for both tests were held by the patients themselves at a distance of approximately 40 cm, with their arms resting on a small table. The room was adequately illuminated and maintained the same illumination in both the initial and final tests. In addition, both tests were performed in a room away from possible sources of noise that could be a distracting factor.

The visual skills test is designed to reflect the efficiency and effectiveness of patients' ocular motility and control (ability to maintain a steady fixation and move their eyes quickly, easily and accurately). These visual skills are basic to fluent and efficient reading. The visual skills tests were composed of two tests administered in succession.

Test 1 Maintenance of fixation (15 s)

The patient was given an A4 size sheet with an X in the centre. He was given the following instructions: “When we tell you to start we need you to look uninterruptedly for 15 s at the X shown on the sheet. Try not to move and try not to move your eyes.” Once the test was completed, the patients were to close eyes. The software used the central 10 s to perform the calculations. The variables obtained in this test were: Variable 1; Fixations and Variable 2; Mean saccade size %.

Test 2 Motility (20 s)

The patient was given an A4 size sheet with two Xs. The following instructions were given: “When we tell you to start we need you to look as quickly as possible at one of the X's and then look at the other one and alternate uninterruptedly for 20 sec.” Once the test was completed, the patients were to close eyes. The software used the central 15 s to perform the calculations. The variables obtained in this test were: Variable 3; Excursions, Variable 4; Fixations and Variable 5; Average duration of fixation.

### Evaluation and scoring of primitive reflexes.

The PRs used in the reviews were four, asymmetric tonic neck reflex (ATNR), symmetric tonic neck reflex (STNR), tonic labyrinthine reflex (TLR) and Moro reflex (MR).

Asymmetrical tonic neck reflex

The patient was placed in a quadruped position with arms straight at shoulder height, both arms and legs were placed perpendicular to the floor and parallel to each other. The therapist held the patient's head while the patient kept his eyes preferably closed or with an eye mask while slowly rotating his head in the direction of one of the sides (Z-axis) until the chin reached the corresponding shoulder. There he held the position for 10 s and then performed the rotation in the opposite direction until the chin reached the position of the opposite shoulder, again holding the position for another 10 s. The operation was repeated four times in total; this test is called Ayres 1. After a brief rest the same sequence of movements was performed again but in this case the patient had to keep the arms slightly flexed for the duration of this test, which is called Ayres 2, and is more demanding. It was scored according to the absence or degree of flexion of the opposite arm. This score was the same for both tests.

A third test only performed on patients older than 5^1/2^ -6 years was the so-called Hoff-Schilder test. The patient was asked to stand in an upright position, barefoot with feet together, head facing forward and arms raised facing forward. The arms were held extended forward and straight with respect to the shoulders, perpendicular to the body and parallel to the floor, while the hands remained relaxed, as if drooping. The patient was again fitted with an eye mask. The therapist held the patient's head by gently rotating it to one side until the chin reached the position of the corresponding shoulder. The therapist held this position for 10 s and then rotated the head to the opposite side until the chin again reached the position of the opposite shoulder, again holding the position for a further 10 s. The patient's head was then rotated to the opposite side until the chin again reached the position of the opposite shoulder. The operation was repeated four times in total. The degree of deviation of the arms was scored according to the rotation of the head (Z-axis).

Symmetrical tonic neck reflex

The patient was placed in a quadruped position with arms straight at shoulder height, both arms and legs were placed perpendicular to the floor and parallel to each other. The therapist held the patient's head while the patient kept the eyes preferably closed or with an eye mask while gently pulling the head as if to look at the ceiling, maintaining this position for 10 s (Y-axis). The head was then pulled in the opposite direction until the chin came to touch the sternum, holding it for another 10 s. The operation was repeated four times in total. It was scored according to the absence or degree of flexion of both arms and/or movement of the trunk (buttocks).

Tonic labyrinthine reflex

The patient stood in an upright position, barefoot with feet together, arms close to the body and looking straight ahead. The instructions given by the therapist were to very slowly and with eyes open flex the head as if he was going to look at the floor trying only to move his head. Once the chin touched the sternum, he was encouraged to remain in this position for 10 seconds. He was then instructed to slowly bring his head to the most posterior position, as if he were going to look at the ceiling, and once reached to hold the position again for another 10 s. The test was repeated four times in total. The whole sequence was then repeated, but this time with the eyes closed. Usually a blindfold designed for these exercises was used to ensure that the patients had zero vision. The therapist always remained in front of the patient to avoid possible falls. The body oscillations appeared both laterally (X-axis) and anteroposteriorly (Y-axis) as well as the compensatory displacement of the feet were scored.

Moro reflex

The patient was in an upright position, barefoot with the feet together, keeping the arms semi-flexed at chest level and with the hands relaxed but not touching. The head was slightly extended and the eyes were covered with a blindfold. The patient was then instructed to maintain the position of the body "like a statue", without moving the legs or arms, and then the therapist gently leaned the patient backwards at an angle of approximately 30º. Once the position was reached, he was told that he was to be quickly and briefly released and then picked up again. To do this, his hands were simply removed from behind his back to allow his body to reach a sudden acceleration and he was quickly re-gripped. This test was attempted only once as it is usually very stressful. We scored especially the no or possible abduction of the arms and secondly the compensatory displacement of the legs.

The score for all reflexes was made based on five variables.

"0" No presence of the reflex. Complete inhibition of the reflex without alteration to the original position.

"1" Residual presence of the reflex. Very subtle alterations or movements from the original position.

"2" Average presence of the reflex. Clear presence with abnormal motor movement or alteration.

"3" Obvious presence of the reflex. There are quite obvious changes associated with it and low motor control.

"4" Retained presence of the reflex. The reflex is observed in its totality and complexity and with all possible alterations present.

The two therapists who performed the motor evaluations are officially trained in both therapies used, so that both the scores as well as the exercises prescribed to the patients were done under the same strict criteria. Therapy ended when the four reflexes indicated above were determined to be practically inhibited; that is, when a maximum of two of these reflexes obtained a residual value of "one" with no apparent possibility of reaching complete inhibition and the remaining two scores at "zero" or when all reached values with scores of "zero". In cases that raised some doubt in the score or other doubts, it was the second therapist who independently reevaluated and scored the patient and determined whether the therapy should continue to try to reach the "zero" value in any of the four PRs or whether it was decided to terminate the therapy because it was determined that no further improvement was possible.

At the end of the study, "Appendix A" has been included which hypothesizes how the PRs play a crucial role in the control of the X, Z and Y motor axes. The concept of homeostasis is described from a motor-functional point of view of the body and in turn how this motor stability transcends to ocular control. The inhibition of the four PRs described in this study directly influences the organization of the body and ultimately translates into improved visual skills. We include a figure that describes these processes in detail.

## Results

The visual skills report provides automatic calculations of the fixation and motility parts of the test. The Visagraph^TM^ III allows, through test routines and automatic calculation, to collect normative behaviors and establish criteria in terms of visual efficiency and competence. SPSS software (version 18.0; SPSS Inc., Chicago, IL, USA) was used for statistical analysis.

This research included 103 males and 90 females ranging in age from 4 to 16 years. The average age of the patients when they started therapy was (M = 8.1, SD = 2.14) and (M = 9.2, SD = 2.09) when they completed therapy. The average period of therapy was (M = 12.2, SD = 2.68), Min. 8 months, Max. 21 months.

Table 1 shows the initial and final scores of each of the tests performed for the four reflexes evaluated. The final scores are significantly lower than those obtained prior to primitive reflex inhibition therapy.

**Table 1.** Scores obtained in the first and last review of primitive reflexes.

	ATNR						STNR		TLR				Moro
	Ayres 1		Ayres 2		Schilder				Open eyes		Closed eyes		
	Left	Right	Left	Right	Left	Right	Head	Head	Head	Head	Head	Head	
Initial Mean	1.76	2.30	2.42	2.71	2.86	2.71	1.37	1.08	1.48	1.76	2.31	2.63	3.64
SD	1.26	1.06	1.14	0.98	0.96	1.07	1.40	1.33	115	1.10	1.01	1.02	0.93
Final Mean	0.10	0.15	0.10	0.17	0.42	0.39	0.12	0.14	0.10	0.11	0.50	0.62	0.30
SD	0.30	0.38	0.31	0.44	0.58	0.65	0.40	0.58	0.35	0.31	0.69	0.75	0.69

Table 2 shows the percentage of patients who showed active reflexes in each of the tests comprising the four primitive reflexes evaluated from a residual score of 1 to a maximum score of 4.

**Table 2.** Percentage of patients with active primitive reflexes at first and last evaluation.

	ATNR						STNR		TLR				Moro
	Ayres 1		Ayres 2		Schilder				Open eyes		Closed eyes		
	Left arm	Right arm	Left arm	Right arm	Left arm	Right arm	Head flexion	Head ext.	Head flexion	Head ext.	Head flexion	Head ext.	
Initial %	80.3	92.7	94.9	98.9	97.8	96.6	61.8	52.5	79.2	87.1	98.9	99.4	96.6
Final %	10.1	13.5	9.0	14.6	37.1	31.5	9.6	8.4	9.0	10.7	42.1	48.9	19.1

Table 3 shows the initial average score (M = 2.23, SD = 0.64) of the 13 measurements collected on the four primitive reflexes measured and the final average of the same measurements (M = 0.24, SD = 0.22) at the end of inhibition therapy. The Wilcoxon pairwise contrast revealed with 95% confidence that primitive reflex inhibition therapy produced a highly significant decrease in the final scores of the four primitive reflexes measured in this test (p < 0.001).

**Table 3.** Average of the initial and final scores obtained in the first and last primitive reflexes review.

	Difference in scores	
	Initial scores	Final scores
Mean	2.23	0.24
SD	0.64	0.22
Wilcoxon test	Final scores - Initial scores	
*p*-value	<0.001	

### TEST 1. FIXATIONS

Variable 1. Maintenance of fixation

The fixation maintenance test assessed the ability of patients to maintain constant fixation on a central X for three frames (15 s) of recording without changing eye position. The central 10 s of time was analysed. A single fixation result for both eyes would be ideal. However, most patients do not achieve this level of performance. The software set the result as acceptable when it measured one or two fixations in both eyes and displayed the message that the patient had difficulty maintaining focus or steady gaze when three or more fixations appeared in either eye.

Calculations represented the total number of fixations (or times the eyes moved a distance greater than 3% of the average global range of eye movement) during 15 s.

All measures revealed strong decreases after PR therapy (see Table 4). The mean OS fixation counts dropped significantly between initial measures (M = 14.08, SD = 14.62) and final measures (M = 4.56, SD = 5.49). The mean OD fixations matched this pattern between initial measures (M = 12.45, SD = 14.22) and final measures (M = 4.42, SD = 5.42). Applying the Wilcoxon pairwise contrast, it could be concluded that PR inhibition significantly decreased the average of the variable fixations in both left and right eyes (*p* < 0.001).

**Table 4.** Results of the fixation maintenance variable (15 s) with the mean values obtained in the left eye (OS) and right eye (OD).

	OS fixations		OD fixations	
	Initial	Final	Initial	Final
Mean	14.08	4.56	12.45	4.42
SD	14.62	5.49	14.22	5.42
Wilcoxon test	OS final - OS initial		Final OD - Initial OD	
*p*-value	<0.001		<0.001	

As a second check on this variable, our interest was to know whether, in addition to significantly reducing the number of fixations, the therapy facilitated a greater balance, interval (-2, +2) between values of both eyes or even perfect balance, the latter being understood as when exactly the same value was obtained in each eye. The Wilcoxon pairwise contrast confirmed a significant improvement (p = 0.005) in the homogeneity of fixations between the initial (M = 1.64, SD = 8.70) and final (M = 0.13, SD = 1.46) fixation measures of both eyes (see Table 5).

**Table 5.** Results of the differences between initial and final fixations in the maintenance of fixation (15 s).

	Difference in fixations	
	Difference initial fixations	Difference final fixations
Mean	1.64	0.13
SD	8.70	1.46
Wilcoxon test	Final fixations - Initial fixations	
*p*-value	0.005	

**Figure 1.**
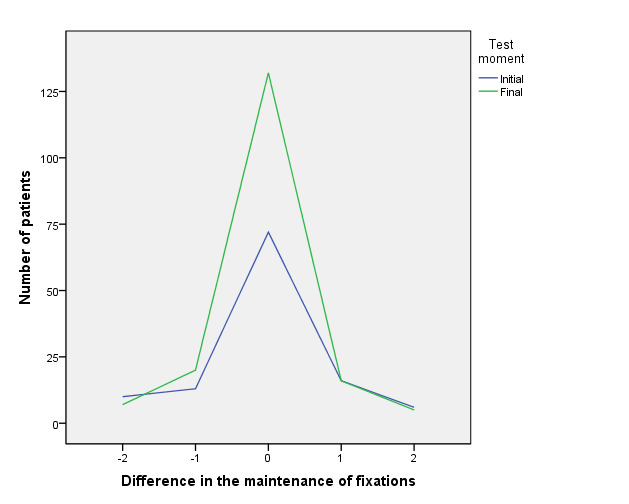
Initial and final comparison of patients with a difference between both eyes of less than 2 fixations in the fixation maintenance test (15 s).

Figure 1 shows the contrast between the initial and final number of patients whose values between both eyes showed a maximum difference of two fixations. At the beginning of therapy 72 patients (37,3%) had perfect balance, with no difference (zero) between both eyes and 55 patients (28,4%) a difference of one or two fixations. At the end of therapy 132 patients (68,4%) achieved perfect balance while 45 patients (23,3%) showed between one or two fixations difference.

**Figure 2.**
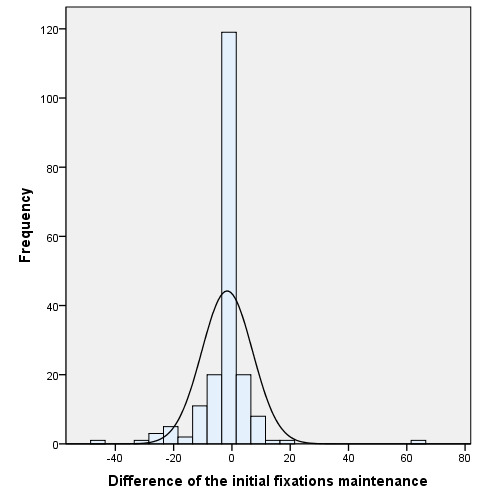

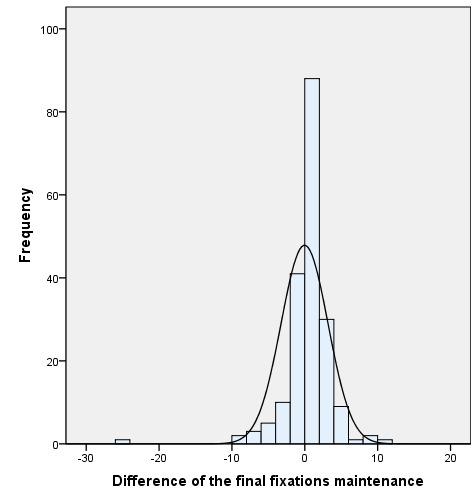
Initial and final comparison of fixations in the fixation holding test (15 s)

Figure 2 shows the difference in all the ranges obtained during the fixation test between the values obtained before and after the therapy.

Variable 2. Mean saccade size %

This parameter measured the mean saccade size during the fixation maintenance test expressed as a percentage of the mean excursions during the motility test. Ideally, patients should be able to direct gaze and maintain fixation on the central X during the 15-s period. A single fixation of both eyes should correspond to 0% mean deviation. However, most patients do not achieve this level of performance. Therefore a value as close to 0% and balanced between both eyes was considered to be the most appropriate.

All measures revealed strong decreases after PR therapy (see Table 6). The mean OS saccade size dropped significantly between initial percentage measures (M = 14.66, SD = 12.34) and final measures (M = 9.32, SD = 8.20). The mean OD saccade size matched this pattern between initial measures (M = 15.64, SD = 16.20) and final measures (M = 11.94, SD = 12.58). Applying the Wilcoxon pairwise contrast, it could be concluded that PR inhibition significantly decreased the mean variable % mean saccade size in both left (p < 0.001) and right eyes (p < 0.007).

**Table 6.** Results of the variable Mean saccade size % with average values obtained in the left eye (OS) and in the right eye (OD).

	OS mean saccade size %		OD mean saccade size %	
	Initial	Final	Initial	Final
Mean	14.66	9.32	15.64	11.94
SD	12.34	8.20	16.20	12.58
Wilcoxon test	OS final - OS initial		OD final - OD initial	
*p*-value	<0.001		0.007	

A second value we obtained was whether independently of the significant reduction of the values in both eyes, to know if the balance between the difference of the values of both eyes before and after therapy was maintained. The mean of the differences between both eyes increased between initial measures (M = -0.98, SD = 13.24) and final measures (M = -2.63, SD = 12.40). However the Wilcoxon pairwise contrast used to compare both differences revealed that the average of the initial and final differences were not significantly different (p = 0.169) (see Table 7).

**Table 7.** Results of the differences between the average size of the initial and final saccades in %.

	Difference in mean saccade size %	
	Initial average	Final average
Mean	-0.98	-2.63
SD	13.24	12.40
Wilcoxon test	Final average - Initial average	
*p*-value	0.169	

### TEST 2. MOTILITY

Variable 3. Excursions

The motility test measured the ability to move the eyes quickly and accurately between two Xs for 4 frames or recording. The number of excursions or saccades was recorded, as well as the accuracy with which they were performed. The patient was instructed to fixate quickly toward one of the Xs and then also as quickly as possible toward the opposite X and so on between the Xs as many times as possible for 20 s. Ideally, a patient should be able to perform a minimum of 30 excursions (left to right or right to left) during the central 15 s analysed. It was therefore desirable to obtain as many excursions as possible in this test. When the minimum value of excursions was not reached, the software displayed a message indicating that the patient has difficulty with ocular motility.

Table 8 shows that the final average of excursions (M = 25.94, SD = 7.38) exceeded the initial average (M = 19.86, SD = 8.69). Paired t-test revealed that excursions increased significantly (p < 0.001).

**Table 8.** Results of excursions performed in Motility test (20 s)

	Excursions difference	
	Initial	Final
Mean	19.86	25.94
SD	8.69	7.38
t-test	Initial-final	
Mean	-6.08	
SD	9.37	
*p*-value	<0.001	

Variable 4. Fixations during saccades

This variable measured the number of fixations the patient made while performing the motility test. Patients with less ocular facility also show more head movements during recording and require more pause time per fixation. If the patient's ability to direct the eyes accurately is limited, compensatory saccadic movements are required, which increases the number of fixations recorded. Although there is no exact number of fixations that determines the ideal value per age or school year, the manufacturer proposes as a guideline a value considered reasonable at two fixations per excursion in a test with a total of 30 excursions. However, the most important aspect is the aspect of equivalence of the number of fixations for the left and right eye and ideally the number of fixations should be the same for both eyes.

Table 9 shows that the mean of this variable increased at the end of therapy (M = 46.27, SD = 11.90) from baseline (M = 43.12, SD = 17.86) in OS. The average in the OD value also increased (M = 46.31, SD = 12.43) with respect to baseline (M = 41.92, SD = 17.13). Paired t-test revealed that the performance of therapy produced a significant increase in the average of this variable in both left (p = 0.013) and right (p = 0.001) eyes.

**Table 9.** Results of the averages obtained in left eye (OS) and right eye (OD) for the variable Fixations during saccade motility (20 s).

	OS excursions fixations		OD excursions fixations	
	Initial	Final	Initial	Final
Mean	43.12	46.27	41.92	46.31
SD	17.86	11.90	17.13	12.43
t-test	Initial-final		Initial-final	
Mean	-3.15		-4.39	
SD	17.54		17.38	
*p*-value	0.013		0.001	

Table 10 shows the difference of the initial average values of OS (M = 43.12, SD = 17.86) and OD (M = 41.92, SD = 17.13) with respect to the difference of the final averages of OS (M = 46.27, SD = 11.90) and OD (M = 46.31, SD = 12.43). The result (M = 0.04, SD = 3.22) revealed with the Wilcoxon pairwise contrast that the average of this variable decreased significantly with the performance of the therapy, which confirmed that it significantly improved the balance between both eyes (p = 0.041).

**Table 10.** Result of the difference of the means of the right eye (OD) and left eye (OS) between the initial and final fixations of the excursions in the motility test (20 s).

	Initial final excursions fixation difference	
	OD-OS Initial difference	OD-OS Final difference
Mean	1.21	0.04
SD	5.74	3.22
Wilcoxon test	Final difference - initial difference	
*p*-value	0.041	

**Figure 3.**
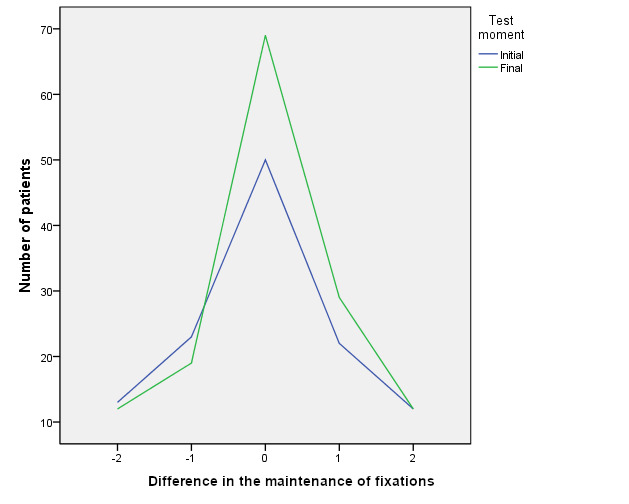
Initial and final comparison of patients with a difference between both eyes of less than two fixations in the motility maintenance test (20 s).

Prior to therapy 50 patients (25.9%) showed perfect fixation balance, which meant an equal number of fixations in both eyes. At the end of therapy the number of patients showing perfect balance increased to 69 patients (35.7%). As for acceptable balance (Difference of fixations within the range -2 to +2), at the beginning of the test 120 patients (62.1%) showed this range compared to 141 (73.1%) at the end of therapy (see Figure 3).

**Figure 4.**
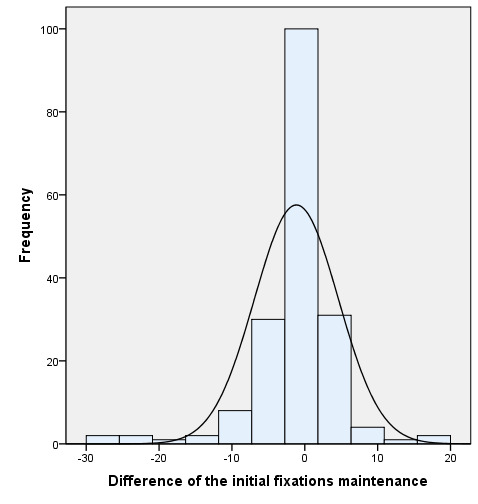

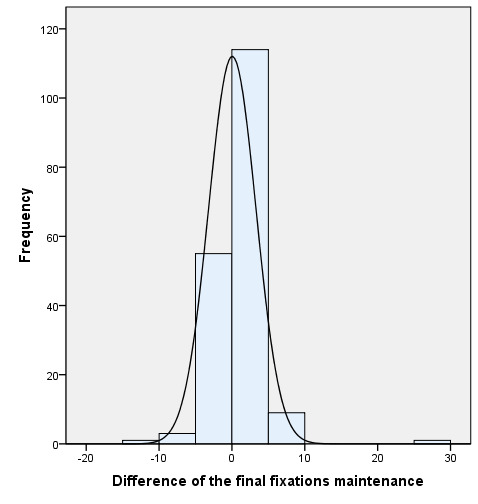
Initial and final comparison of the difference of fixations of the motility variable (20 s).

Figure 4 shows how the difference in fixations between the two eyes during the motility test was balanced, reducing the difference from the beginning to the end of the therapy.

Although the number of fixations during motility testing increased, this should be calculated as the ratio of the increase in fixations to the increase in excursions. As can be seen in Table 11, the mean OS number of fixations per excursion dropped significantly between initial measures (M = 2.16, SD = 0.89) and final measures (M = 1.54, SD = 0.40). The mean OD fixations per excursion matched this pattern between initial measures (M = 2.10, SD = 0.86) and final measures (M = 1.54, SD = 0.41). Paired t-test (p < 0.001) revealed a significant decrease in both eyes in the mean number of fixations per excursion at the end of PR therapy (p < 0.001).

**Table 11.** Results with the evolution of the average of the initial and final fixation variables per excursion in the left eye (OS) and in the right eye (OD).

	Mean of fixation/excursion in motility test			
	OS initial	OS final	OD initial	OD final
Mean	2.16	1.54	2.10	1.54
SD	0.89	0.40	0.86	0.41
t-test	Final-initial		Final-initial	
Mean	-0.61		-0.55	
*p*-value	<0.001		<0.001	

Table 12 shows that with the results obtained, a final mean value (M = 0.0012, SD = 0.11) was observed, significantly lower than at baseline (M = 0.0604, SD = 0.29). The p-value of the Wilcoxon contrast revealed that the performance of the therapy succeeded in increasing the balance between the eyes, since it significantly decreased the difference between the measures of this variable (p = 0*.*023).

**Table 12.** Results with the evolution of the equilibrium of the average of the initial and final left (OS) and right eye (OD) fixation variables by excursion.

	Difference in average fixation per excursion	
	OS initial - OD initial	OS final - OD final
Mean	0.0604	0.0012
SD	0.29	0.11
Wilcoxon test	(OS final - OD final) - (OS initial - OD initial)	
*p*-value	0.023	

**Figure 5.**
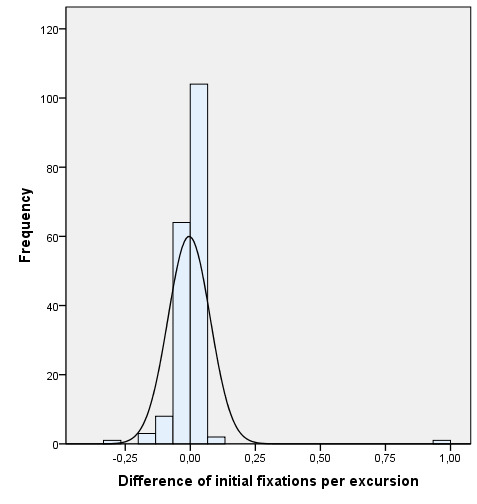

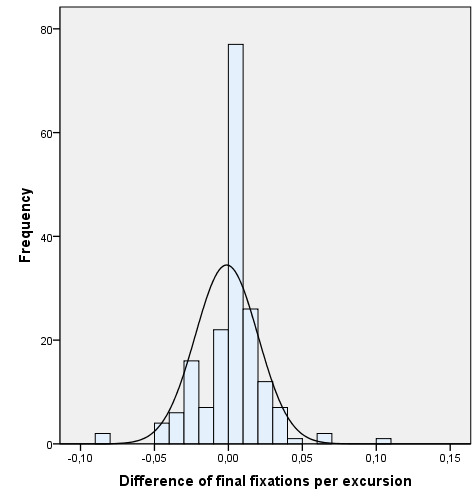
Comparison between the difference of the initial average and the difference of the final average of fixations per excursion during the motility test (20 s).

Figure 5 shows that the difference in the average between the two eyes of the fixations per excursion from the beginning to the end of the therapy is significantly reduced.

Variable 5. Average duration of fixation

This variable measured the average time that both eyes maintained fixation on each of the Xs while performing the motility test. Although there is no exact number of mean fixation duration that determines the ideal value per age or school year, the manufacturer proposes as a guideline a value considered reasonable, which is 0.35 s per fixation in a test with a total number of 30 excursions. It is very important that the value for both eyes is as similar as possible.

Table 13 shows the initial average values of OS (M = 0.3531, SD = 0.12) and OD (M = 0.3577, SD = 0.10) with respect to the final averages of OS (M = 0.3371, SD = 0.08) and OD (M = 0.3383, SD = 0.08). The Wilcoxon pairwise contrast revealed that therapy significantly reduced the averages of fixation duration in both eyes from baseline to end of therapy, as both p-values are less than 0.10 twice the significance 0.05, because it is a one-sided contrast.

**Table 13.** Results of Average left (OS) and right eye (OD) fixations duration.

	OS/OD average duration of fixation			
	OS initial	OD initial	OS final	OD final
Mean	0.3531	0.3577	0.3371	0.3383
SD	0.12	0.10	0.08	0.08
Wilcoxon test	OS final - OS initial		OS final - OS initial	
*p*-value	0.083		0.025	

Table 14 shows the initial average difference value (M = 0.0046, SD = 0.08) of OS (M = 0.3531, SD = 0.12) and OD (M = 0.3577, SD = 0.10) with respect to the final average difference value (M = 0.0012, SD = 0.02) of OS (M = 0.3371, SD = 0.08) and OD (M = 0.3383, SD = 0.08). The pairwise Wilcoxon test revealed that the final average difference in fixation duration was significantly less than the initial average difference, confirming that there was a significant improvement in inter-eye balance with therapy, as p-value is less than 0.10 twice the significance 0.05, because it is a one-sided contrast.

**Table 14.** Results of the difference between the left eye (OS) and right eye (OD) averages of the initial and final fixation durations.

OS/OD average duration of fixation		
	Initial	Final
Mean	0.0046	0.0012
SD	0.08	0.02
Wilcoxon test	Final average difference - initial average difference	
*p*-value	0.075	

As a last approach we tried to verify whether the average values of fixation duration were linked to the initial and final excursions. At both the initial and final moments of the test, the values obtained in the Pearson pairwise correlation revealed with a confidence level of 99%, that as the values of the excursion variables increased, the values of the average duration fixation variables decreased very significantly (p < 0.001) see Table 15.

**Table 15.** Results of the relationship between the duration of left eye (OS) and right eye (OD) bindings and excursions.

Average duration fixation				
	Initial excursions		Final excursions	
	OS initial	OD initial	OS final	OD final
Pearson's r	-0.32	-0.41	-0.51	-0.51
*p*-value	<0.001	<0.001	<0.001	<0.001

**Figure 6.**
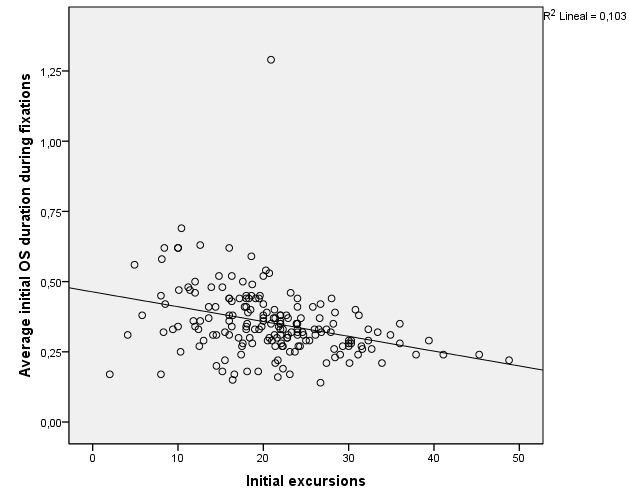

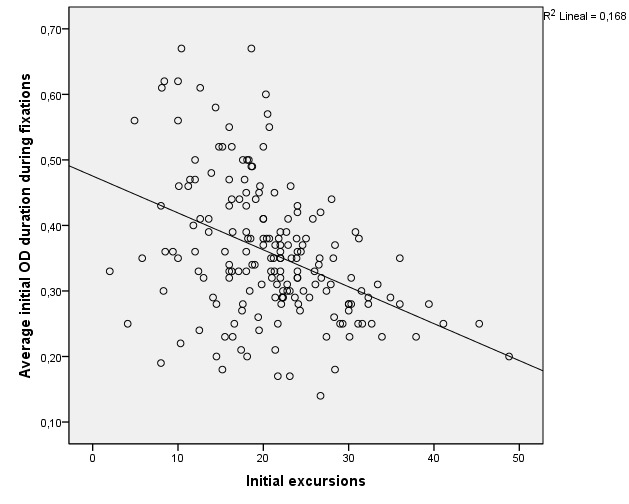

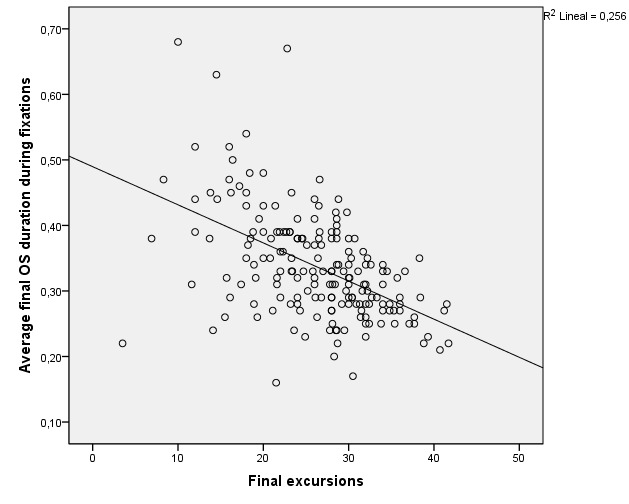

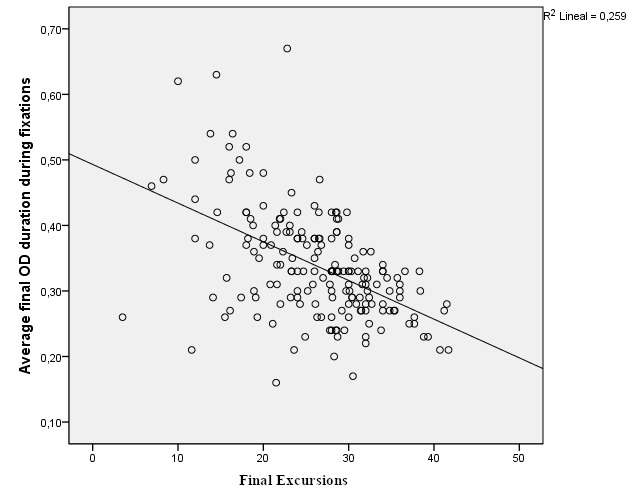
Relationship between the behavior of the excursion value and the mean fixation duration of the left eye (OS) and the right eye (OD) before and after motor therapy.

Figure 6 shows the scatter diagrams of the four variables corresponding to the Average duration fixation with respect to the final and initial excursions, allowing us to visualize their statistical or approximate dependence. Both the shape of the clouds and the slope of the regression lines confirm that the dependence is negative or inverse, as demonstrated above. The coefficients of determination R-squared inform us of the appropriate adjustments given the sample size.

Table 16 revealed results on the possible inverse relationship between more active PRs and lower levels of visual skills. Individually, the best reflex that correlated with visual skills was the TLR, both at the beginning and at the end of the treatment. The results revealed that the lower its presence, the better the visual skills, and vice versa. Subsequently, the ATNR behaved almost identically. The STNR showed some correlation, although not significant overall, while the MR showed absolute independence. In terms of average/group, at baseline, reflex scores were positively and very significantly correlated with initial fixations, as measured by the fixation maintenance test (rs = 0.318, p < 0.001). It could be observed that as the values of the reflexes improved, decreasing from a score of 4, which is the least desirable, towards a score of 0, which is the most desirable, the fixations also improved, descending from high values towards 1, where one fixation represented the best possible outcome. The retained presence of a PR is determined by a value of 4, whereas a value of 0 represents the inhibition of a PR and thus its absence. As for visual fixations, a value of 3 fixations or higher was already considered inadequate. Similarly, there was a highly significant correlation with initial excursions, albeit negative (rs = -0.241, p = 0.001), which meant that as reflexes improved, the excursions also improved, increasing toward higher values. For this test to be considered the most adequate, the patient had to perform a minimum of thirty excursions. At the final moment, reflex scores were correlated, although not significantly, with final fixations (rs = 0.136, p = 0.071). On the contrary, with the final excursions (rs = -0.233, p = 0.002), it could be concluded that given the negative sign of the correlation coefficient, as the final reflexes improved and their scores decreased, the final excursions improved significantly as their value increased.

**Table 16.** Relationship between PR scores and visual skill levels.

		Average of the initial fixations	Average of the final fixations	Average of the initial excursions	Average of the final excursions
Average of the 4 PRs	Spearman *r*_s_	0.318	0.136	-0.241	-0.233
	*p*-value	<0.001	0.071	0.001	0.002
ATNR	Spearman *r*_s_	0,250	0,042	-0,191	-0,202
	*p*-value	0,001	0,573	0,011	0,007
STNR	Spearman *r*_s_	0,258	0,052	-0,125	-0,133
	*p*-value	0,001	0,493	0,095	0,077
TLR	Spearman *r*_s_	0,212	0,125	-0,244	-0,170
	*p*-value	0,005	0,095	0,001	0,023
MR	Spearman *r*_s_	0,082	-0,001	-0,003	-0,076
	*p*-value	0,278	0,992	0,970	0,316

## Discussion

Inhibition of the four relevant PRs, ATNR, STNR, TLR and MR facilitated an improvement in visual skills such as visual fixation by significantly reducing the eye movements required to maintain attention and also improved saccadic movements by significantly increasing them at the end of therapy. Balance is another of the factors underlying the stimulation, since except for one variable in which the values were significantly altered, although not significantly, the rest of the variables showed a significant improvement in the balance between the values of both eyes. Thus, an obvious conclusion is that this improvement makes eye movements more precise, easier, more controlled and with a more efficient oculomotor system, since the movements are performed with less effort. This effect provides a solid base for future tasks such as attention or reading processes. Finally, we could propose that the retained presence of at least these four PRs could be an evident cause of asymmetries or alterations in ocular fixation and motility values.

### PRIMITIVE REFLEXES

The scores performed on each patient initially yielded very high values, as would be expected. The reflex that initially remained by far the most active was the MR, followed by the ATNR in its Ayres 1 variant and then in its Ayres 2 variant. However, after performing the inhibition therapy of the PRs, the most complex reflex to inhibit was the TLR in both flexion and extension in the test performed with the eyes closed. Although the highest initial score was found in the MR, it was reduced below other reflexes whose initial scores remained below it. In general terms the averages of all final scores (M = 0.24, SD = 0.22) remained below the value 1, considered the score that determines a residual presence of a reflex. The average value of the mean scores at baseline was (M = 2.23, SD = 0.64) on a maximum scale of 4. The process of the PR inhibition after an average period of (M = 12.2, SD = 2.68) months concluded with a highly significant decrease in all measured parameters.

The data obtained also showed that the initial highest permanency of the PRs in terms of percentage were obtained in the TLR with eyes closed, followed by the Schilder test/Ayres 2 and MR, which again highlights the difficulty that both TLR as Schilder Test showed in their inhibition. It should be noted that although the presence of certain reflexes remained high in percentage terms, the scores were residual.

The results revealed that the highly significant reduction in the mean values of all the measures comprising the four PRs measured in this research were accompanied by a highly significant improvement in the ocular values.

### TEST 1. FIXATIONS

Variable 1. Maintenance of fixation

This test is designed to assess the subject's ability to focus and control attention and maintain binocular fusion for a period of time that exceeds the usual reading requirements. As indicated the ideal value is to perform one fixation in each eye, with the maximum value interpreted as adequate being two fixations in both eyes. If there are multiple fixations, starting with three fixations, but the number is the same for both eyes, this would suggest difficulty with one or more of the following:

- Failed to maintain attention on task.

- Vergence (binocular fusion at a common fixation point) could not be maintained and had to be restored at intervals.

The habitual tendency to keep the eyes moving caused involuntary re-fixations.

However, it can be specified that up to five fixations can be common, causing the difficulties mentioned above. These difficulties become more acute with a higher number of fixations.

A more important consideration was any difference in the number of fixations between the two eyes. While a difference of one or two fixations between the two eyes might not be significant, a larger difference might indicate a lack of binocular control. The variations can range from a single fixation to as many as twenty fixations or more. The result of the investigation showed that although the final average did not reach a maximum of two fixations, they did decrease significantly to (M = 4.56, SD = 5.49) in the OS, which was a decrease of 68% and (M = 4.42, SD = 5.42) in the OD which was a decrease of 64%. The number of patients who initially presented perfect balance between both eyes increased from 37.3% before therapy to 68.4% after therapy. In the case of acceptable balance, the range of -2 to +2 fixations difference between both eyes went from 70.9% before therapy to 93.2% after the end of therapy. This homogeneity could mean an improvement in binocular fusion, a decrease in involuntary refixations and an improvement in the maintenance of attention.

Variable 2. Mean saccade size %

This parameter provides information on the average saccade size expressed in % while performing the fixation test. A value of zero or as close to zero as possible is expected, although it is very difficult to achieve. The sample result confirmed the significant decrease in mean saccade size in both eyes from the initial 14.7% in the OS to 9.3% in the final test, a decrease of 5.4%, while in the OD it went from 15.6% in the initial test to 11.9%, a decrease of 3.7%. However, the difference in the number of fixations between the two eyes is also of great importance. While a difference of one or two fixations between the two eyes may not be significant, a larger difference may indicate a lack of binocular control. An examination of the original chart would probably confirm such a loss of binocular control. As a consequence of this difference, the percentage of mean saccadic size must be taken into account. If this percentage were equal for both eyes, it is likely that the two eyes would simply have changed position. If, on the other hand, there was a difference between the mean saccade size between the two eyes, this would suggest a difficulty with binocular control or fusion. In perspective, a 3% difference between the eyes would be roughly equivalent to a difference of two letter spaces. In some cases where one eye deviates by 7% and the other by 1%, this would equate to a difference of three letter spaces in the fixation position in reading, which would likely indicate a loss of vergence and proper fusion. However, in connection with this hypothesis, it should be noted that individuals vary considerably in their vision ability and report unique vision with varying amounts of retinal disparity, so it can only be stated that the closer this mean saccadic percentage of one eye is to the other, the better. It is likely that a difference in the % mean saccadic size of one eye relative to the other greater than a value of 2-3% could be significant. Pearson's coefficients of variation were 531% in the initial test and 112% in the final test, both of which were very high, although the reduction to one-fifth in dispersion also indicated the usefulness of the therapy in terms of achieving more homogeneity in the balance between eyes. The results revealed that the initial difference was 0.98%, while the final value was 2.63%, reaching the limiting range but not exceeding it. In percentage terms, the increase of 1.65% (2.63%-0.98%) revealed an increase of 168%, although statistically it did not represent a significant increase. This was evident from the calculations obtained, which showed that despite the increase, the averages of the initial and final differences were not significantly different. However, this influence on the visual system may be uneven in certain aspects and although the results in absolute terms are clearly positive, there may be some aspects that need a more concise treatment to correct them, being in this case visual therapy. We believe that the improvements are in general very manifest in mitigating certain minor aspects.

### TEST 2. MOTILITY

Variable 3. Excursions

This task is designed to assess the subject's oculomotor facility (ability to turn the eyes easily and quickly), binocular ability (ability to maintain vergence and fusion) and tracking accuracy (ability to direct the eyes and fixate accurately).

The minimum number of excursions is set at thirty. Typically, fewer excursions occur if the child needs several saccadic movements to execute such wide eye sweeps (15 degrees at a viewing distance of 18 inches) and a longer pause time for each fixation. Therefore, it is important to note the general way in which these excursions were executed, as well as the number performed. The final mean value of the excursions increased significantly from (M = 19.86, SD = 8.69) to (M = 25.94, SD = 7.38) which represented an increase of 30.6%. Patients did not reach the recommended minimum thirty excursions, although this improvement evidenced that patients achieved higher ocular facility, better binocular control and greater accuracy at follow-up.

Variable 4. Fixations during motility test

This variable shows the fixations that each eye performs during the motility test. In the results obtained, there was a significant increase in the values of both eyes in the final test, which is logical since the increase in the total number of excursions with respect to the initial test should also lead to a parallel increase in the fixations in the final test. The average of the fixations increased in the final test by 7.4% in the OS, initial value (M = 43.12, SD = 17.86) to (M = 46.27, SD = 11.90), while in the OD the final increase was 10.5%, initial value (M = 41.92, SD = 17.13) to (M = 46.31, SD = 12.43), this rise being significant in both eyes.

However, in order to reliably study the behaviour of fixations during the motility test, it was also necessary to consider the significant increase in the number of excursions performed at the end of the therapy compared to the excursions performed in the initial test as well as their relevant fixations. The manual approximately indicates for this variable that the fixations which should be measured for each of the excursions performed is set at two fixations per excursion. The results obtained from the fixations for each of the excursions performed at the end of the therapy showed a significant reduction in the final values of both eyes. The initial fixations were (M = 2.16, SD = 0.89) reaching (M = 1.54, SD = 0.40) at the end of therapy in the OS, a decrease of 28.7%. Regarding the OD the initial fixations went from (M = 2.10, SD = 0.86) to (M = 1.54, SD = 0.41) at the end of therapy, which was a decrease of 26.7%. Considering that a patient with poor visual performance could achieve in this test eight excursions with possibly three fixations on average per excursion (manufacturer's data), it could be deduced that with worse visual skills the number of fixations per excursion would be higher. Applying this reasoning the effort patients had to make to perform this test after therapy was less, as patients were able to significantly increase excursions with less effort as they required fewer fixations per excursion, leading to better tracking accuracy.

Additionally, perhaps an even more important parameter is the equivalence of fixations between the two eyes. It should be noted that a difference of one or two fixations is not unusual and may not be significant. However, a difference of considerably more than three fixations may suggest inadequate coordination. For example, poor performance might show a difference of nine or ten fixations between the two eyes. This would most likely suggest very poor binocular coordination ability. The final averages showed practically the same value for both eyes, 46.27 fixations on average for the OI and 46.31 for the OD, achieving complete homogeneity as the coefficients of variation were reduced by 39.5% and 34.1% in OS and OD respectively. The average difference in the initial test of fixations during the motility test was (M = 1.21, SD = 5.74) versus (M = 0.04, SD = 3.22) in the final test, significantly decreasing the average differences between fixations in the motility test. The results also yielded even better conclusions when comparing the balance between eyes with respect to fixations per excursion in the motility test using the difference in averages between OS and OD at baseline and end of therapy. Initial value (M = 0.0604, SD = 0.29) with respect to the final value (M = 0.0012, SD = 0.11), exceeding the initial value at the end by almost doubling it. The performance of the therapy significantly decreased the difference in the average number of fixations per excursion.

At the beginning of the therapy, the patients who showed a perfect balance in fixations between both eyes, considering this balance when the values are the same in both eyes, was 25.9% at the beginning, reaching 35.8% at the end of the therapy; an increase of 9.9%. Regarding the patients who showed an acceptable range of balance at the beginning of the test, values ranging from -2 to +2 fixations difference, it was 62.1%, reaching 73.1% in the final test; an increase of 10.9%.

Obviously, a child with better oculomotor facility, binocular control, and tracking accuracy would have greater potential for good visual/functional performance in reading. Excessive head movement, limited excursions, and greater disparity in the performance of the two eyes would suggest that a child with these characteristics would experience more difficulty in the usual requirements of close reading and that this limited competence would affect the ease and comfort of reading for this child. Beyond any numerical data, the graph obtained allows us to examine oculomotor performance in terms of binocular fusion and coordination. The values as a whole tend to show an improvement in visual performance.

Variable 5. Average duration of fixation.

This parameter measures individually the average time during which both eyes maintain fixation on each of the Xs while performing the saccadic movements in the motility test. An excellent motility performance would be 45 to 50 excursions possibly with an average duration per fixation of approximately 0.30 seconds. A very poor performance could be as low as eight excursions or less, with a very long fixation duration, possibly around 0.58 seconds. The results show a decrease of the average in OS, initial value (M = 0.3531, SD = 0.12) and final value (M = 0.3371, SD = 0.08) s representing a decrease of 4.5%. As for the OD, initial value (M = 0.3577, SD = 0.10) versus final value (M = 0.3383, SD = 0.08) s, representing a reduction of 1.9%. Although the percentage may seem low, the result clearly shows a significant improvement in the average fixation time at the end of therapy. As with the other variables, there is an interest in knowing whether the mean duration per fixation also improved its balance between both eyes. The average mean time per fixation was (M = -0.0046, SD = 0.08) at the beginning of the test and (M = -0.0012, SD = 0.02) at the end of the test, resulting in a significant reduction of the average duration of fixations in both eyes at the end of therapy.

Finally, the average duration of fixations was compared with respect to the number of excursions obtained both at the beginning and at the end of the test. The initial average per excursion for OS was -0.32 and -0.41 for OD. The final average for OS was -0.51 and -0.51 for OD. The result significantly revealed an inverse relationship between excursions and average fixation time per excursion, so that the more saccades in the motility test, the shorter the mean time taken to maintain fixation in each eye. This result evidenced that in order to perform more excursions the time that both eyes have to remain on fixation must be shorter.

Among the patients who met the inclusion criteria, there were two cases in which all measurements in the initial visual skills test resulted in a value of zero in each and every one of the variables. In these cases the test was repeated up to two times with maximum readjustment of the interpupillary distance, and it was impossible for the device to show any measurement. In these two cases the final measurements, after the end of the therapy, were successful at the first attempt in each and every one of the variables measured. Also in the initial test, ten other patients were observed in which one, two or even three variables showed a measurement value of zero. These were considered valid because in all cases a minimum of two of the five variables measured with data readout appeared. In those same ten patients the final measurements, after PR therapy, were obtained on the first attempt without any difficulty and with no variable with a value of zero. We believe that the cases described above were due to an inability of the device to perform measurements as they were possibly out of range or perhaps exceeded the ceiling limit values considered reasonable, although we believe that the impossibility in the ocular measurement was perhaps due to punctual deviations of one of the eyes during the test or part of the test, the latter possibility being the most probable. In the opposite case, only one case was obtained in which the device read all the variables during the initial test, but failed to detect three of the five variables in the final test. In this case the values of the initial variables showed some of the highest values of the whole series, being clearly reduced in the two final variables that were measured. We believe that possibly the three remaining variables remained still very high or a punctual ocular deviation appeared. This case represented only 0.5% of the final total patients.

After reviewing all the published literature on PRs and the Visagraph^TM^ we did not find any studies correlating these two topics. We did locate 25 publications (1; 2; 11; 15; 17; 19; 23; 30; 33; 36; 40; 43; 44; 48; 49; 50; 51; 53; 57; 58; 61; 64; 66, 67; 68) that analysed the different behaviour of the visual system comparing different reading media, DEM tests, visual syndromes, reading assessment, ADHD, etc. and in which the Visagraph^TM^ III was used for research purposes, but none of the studies sought to test the change that PRs produced on the visual system. Possibly the study with the most similarities to the one presented here was the one conducted by McPhillips et al. (38). In this study, four PRs were stimulated but using an ocular recording device called Ober2, and the result of the investigation showed clinically significant advances in reading after a program of PR inhibition. These data suggest results in the same direction as this study, as the reorganization of the visual system creates the basis for, among other things, the improvement of reading skills.

### Limitations

Being a retrospective research, it could be thought that the chronological growth of the human being itself favours a clear and evident improvement in visual skills. At all levels of efficiency, the greatest changes from school year to school year in reading-related eye movements are observed in the elementary grades (6-11 years). Most eye movement measures tend to stabilize in middle school (12-15 years), while in high school changes in reading-related eye movement measures tend to be modest (16-18 years) (58). It is found that the majority of measurements obtained initially both by age or sex did not reach the values considered ideal in each of the variables. In addition, there were no values specific to each age that should progressively improve as they advanced chronologically, with different values staggered and specific in each range, but on the contrary, the values considered as adequate or ideal were commonly established for all patients whether they attended first grade of primary school or ninth grade of secondary school, when the age difference between the two could be at least 8 years. The result of this paper showed that improvements occurred in all age and sex groups. It should not be ruled out that physiological growth itself acts as a driving mechanism for differences in eye movement behaviour (60), but stimulation of the central nervous system through inhibition of the PRs is likely to organize and accelerate the maturational process. Age-related changes in eye movements during reading are largely a consequence of a maturational process such as sensorimotor control. Also the accumulation of reading experience is important, however readers with low reading skills require greater cognitive effort and such ongoing effort can lead to attentional drift (58). Another aspect that could potentially influence the final results is the relationship exerted by the frontal cortex on auditory spatial attention. Auditory and auditory-motor neurons are modulated by the engagement of visual attention. This implies that fifty percent of auditory neurons show a significant decrease in activation activity when auditory stimuli are presented while executing the visual fixation task (32). The Prefrontal Cortex-BasalGanglia-Thalamic pathway enables selection between vision and audition by suppressing mainly the distracting modality. This pathway also enhances sensory discrimination and is used for suppression of target-directed background noise (41). We do not know how the possible influence of background noise might alter visual abilities compared to the data presented in this paper, although to avoid this effect, the room where the visual tests were performed was preserved as much as possible from any auditory contamination. This is not the case at the school level where it is obviously not possible to isolate the background noise and we do not know what short or long term effects this might have. Nor do we know whether the improvements observed in this paper could be extrapolated to other developmental stages, taking into account a similar transition with a range of 12 years in the age difference between the youngest and oldest patients in the study. This would require a cross-sectional research addressing the approach in a late youth, adulthood, or older population.

Another limitation is the duration of the tests. The tests used required a relatively short period for their evaluation. In school practice, tests tend to be long, with longer attention spans and more complex demands with each new course that begins. They require longer periods of attention, speed and concentration. We believe that the improvements in visual skills will facilitate adaptation to the new school levels, but it is possible that an adaptation with other types of tests may be necessary to verify whether the visual improvements achieved and demonstrated in this paper also respond to longer periods of work.

From the study results, it was possible to determine that of the four PRs evaluated, we could venture that TLR would be the reflex that could best determine the behavior of visual skills from its inhibition since its values were identical to the mean values obtained for all the PRs. The ATNR could also be used for the same purpose, except that at the final moment, the improvement of fixations would show total independence. The STNR showed, at the initial moment, a significant correlation in terms of fixations, while there was a correlation, although not significant, with excursions. At the final moment, it showed complete independence of fixations and a correlation, although not significant, with excursions. Finally, the MR showed absolute independence at both moments with respect to both visual abilities, being the least predictive reflex. In general terms, we can confirm that the greater the presence of initial active reflexes, the worse the visual skills, and vice versa. Thus, when improving the PR scores, there was a significant decrease in fixations at the initial moment and a greater increase in excursions. At the final moment, there was a decrease in fixations, although not significant, while excursions increased significantly. As for visual skills, visual excursions correlated with the PR scores better than visual fixations.

### Conclusion

Although present at the beginning of life and with a clear objective such as helping the neonate in the first survival movements, the PRs may be present beyond three years of life, altering certain visual abilities. The inhibition of the asymmetric tonic neck reflex, symmetric tonic neck reflex, labyrinthine tonic reflex and Moro reflex significantly improve, independently of age and sex, visual fixation, and saccadic movements, favoring tasks with less effort and improving the balance between the values of both eyes. This progress establishes the basis for other more complex processes such as reading. The present research aims to provide valuable information that will help vision professionals and physiological therapists, among others, to obtain new work approaches.

### Ethics and Conflict of Interest

The author declares that the contents of the article are in agreement with the ethics described in http://biblio.unibe.ch/portale/elibrary/BOP/jemr/ethics.html and that there is no conflict of interest regarding the publication of this paper.

### Acknowledgements

We wish to thank Patti Andrich of The Vision Development Team for her advice, Julia Peris-Ferrer and Vicente Vidal-Giner for their help with the graphic design and Professor Antonio Carrillo-Espartero of Centro de Optometría Internacional, for the statistical development. We would also like to thank the entire team at Centro Optométrico Montrull for their help with data collection and especially to Mª Teresa Montrull-Perales for her unconditional support.

## Appendix A

### Body Axes

Hypothesis of body axes and their relation to PRs

All humans seek a position of homeostasis with their environment, a "steady state". Our bodies operate on a system of coordinates and axes of rotation (28).

X-axis. TLR inhibition likely creates better body stability and reduction of oscillatory movements that occur laterally. Although oscillatory movements occur in all directions, lateral oscillations are usually the most common in a non-integrated TLR. Regions corresponding to the body axis receive somatosensory signals that always extend across the midline. Visual and vestibular input also converge in the area corresponding to the body axis. Vestibular input, which also reaches the body axis regions of the motor-sensory cortex by signaling the effect of the force of gravity, provides directionality to the axis (14). Thus the presence of TLR is specifically associated with impaired saccadic eye movement accuracy as well as impaired reading ability (25). Vestibular nuclei are estimated to be involved in adjusting control systems for stable visual fixation (70) and certain attentional disorders are shown to be related to compromised vision and balance problems (4).

Z-axis. The ATNR is possibly responsible for reorganizing and providing the understanding of the vertical midline, crucial in the visual processes of convergence. This axis is also part of the necessary framework for the proper functioning of the lateral vestibulo-ocular reflexes. It is very likely that its inhibition facilitates the improvement of all eye movements related to accommodation and therefore focusing. In fact regarding the motor control of the eyes performed by the cerebellum, there is evidence supporting an involvement of the midline cerebellum, crucial for saccade generation (32) Quite possibly a persistent ATNR directly interferes with saccadic eye movements and alters visual tracking abilities (37).

Y-axis. This axis is most likely to be influenced by two reflexes, the STNR and the TLR, although it is the STNR that exerts the most influence, as it prevents visual stability while being held. It is very likely that the vertical vestibulo-ocular reflexes are affected as well as the control of rapid focus changes. It also has implications for anteroposterior TLR correction and stability. Children with active STNR may have problems with fluency in activities that require shifting the eyes in a vertical line (45).

Although the Moro reflex has a vestibular basis in its activation, it is possible that it interferes reciprocally in the control of vestibular stability, although to a lesser extent. Nevertheless, we believe that this reflex precedes the visual balance necessary for proper focusing at different distances, and its inhibition is critical. Its inhibition facilitates a decrease in sympathetic activity. Sympathetic activation is associated with blurred vision and ocular symptoms such as narrow visual fields and other visual field defects such as oculomotor function dysfunction (24). In the same sense the pupil is controlled by a sphincter and a pupillary dilator, and this is also affected by not entirely visual factors such as alertness and cognitive load (35), so a rapid adaptation of the Parasympathetic Nervous System at basal level is relevant in the behavior of good cognitive performers (21). In already more extreme cases, stressors, either chronic or acute, can lead to a change in the cognitive interpretation of the brain, potentially leading to a loss of the visual field. Activation of the sympathetic nervous system can have effects on vision, and the autonomic system must be rebalanced by reducing the activity of the sympathetic nervous system and activating the parasympathetic one (54).
